# Supplementary material for: Comparative Study of Different Acidic Surface Structures in Solid Catalysts Applied for the Isobutene Dimerization Reaction
Source: Nanomaterials (Basel). 2020 Jun 25;10(6):1235. doi: 10.3390/nano10061235 (PMC7353098; doi:10.3390/nano10061235)
Supplement: Supplementary file 1 [file nanomaterials-10-01235-s001.pdf]

Supplementary Material

Comparative Study of Different Acidic Surface Structures in Solid Catalysts Applied for the Isobutene Dimerization Reaction

José M. Fernández-Morales [1], Eva Castillejos [2,*], Esther Asedegbega-Nieto [1,*], Ana Belén Dongil [3], Inmaculada Rodríguez-Ramos [3] and Antonio Guerrero-Ruiz [1]

[1] Dpto. Química Inorgánica y Técnica, Facultad de Ciencias, UNED, c/Senda del Rey No. 9, 28040 Madrid, Spain; jmfernandez@ccia.uned.es (J.M.F.-M.); aguerrero@ccia.uned.es (A.G.-R.)
[2] Dpto. Ingeniería Química, Facultad de Ciencias, UCM, Avda. Complutense s/n, 28040 Madrid, Spain
[3] Instituto de Catálisis y Petroleoquímica, CSIC, c/Marie Curie No. 2, Cantoblanco, 28049 Madrid, Spain; a.dongil@csic.es (A.B.D.); irodriguez@icp.csic.es (I.R.-R.)
* Correspondence: castillejoseva@ccia.uned.es (E.C.); easedegbega@ccia.uned.es (E.A.-N.)

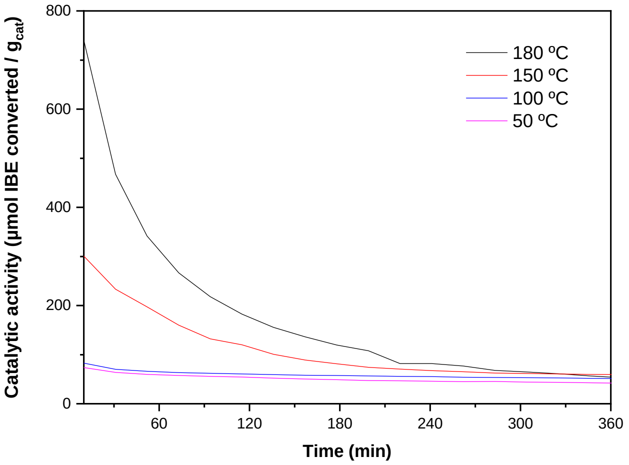

(a)

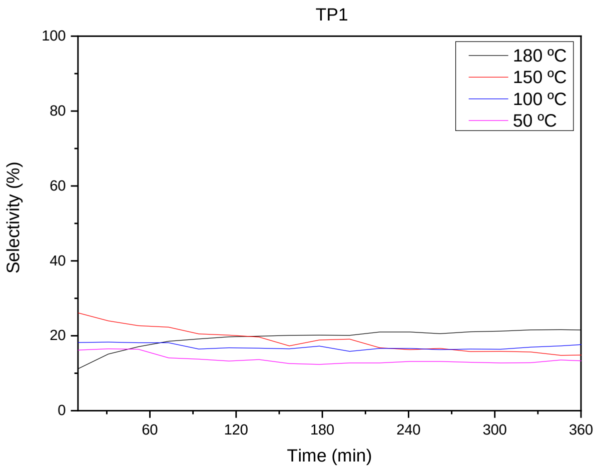

(b)

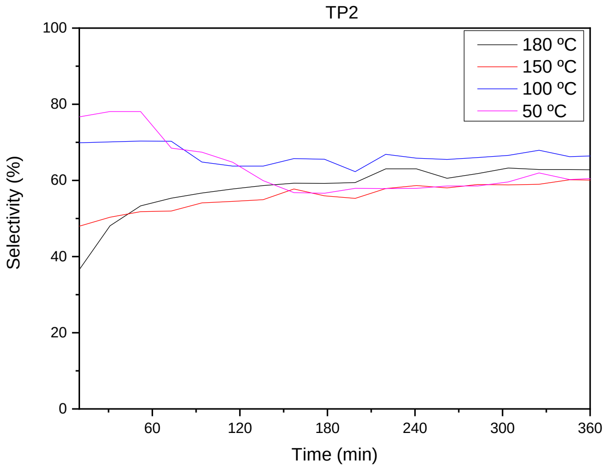

(c)

Figure S1 a) Catalytic activity at 50, 100, 150 and 180 °C for the Amberlyst; b) Selectivity to TP2 and c) to TP1 olefins formed in the IBE dimerization reaction.
